# Supplementary material for: Is Breast Cancer Risk Associated with Menopausal Hormone Therapy Modified by Current or Early Adulthood BMI or Age of First Pregnancy?
Source: Cancers (Basel). 2021 May 31;13(11):2710. doi: 10.3390/cancers13112710 (PMC8199436; doi:10.3390/cancers13112710)
Supplement: Supplementary file 1 [file cancers-13-02710-s001.zip › Table_S1.pdf]

Table S1a HRT status (model without HRT*BMI/BMI20)	N%BC/N% no BC	HR (95% CI)
**Never**	998 (2.8)/ 34397 (97.2)	1.00
**Former**	507 (3.2)/ 15366 (96.8)	1.01 (0.89-1.13)
**Current**	158 (3.6)/ 4182 (96.4)	1.33 (1.12-1.58)
Age		1.02 (1.01-1.03)
BMI		1.18 (1.12-1.24)
Height		1.06 (1.03-1.11)
BMI20		0.77 (0.69-0.86)
BMI*BMI20		0.97 (0.91-1.03)
Age at first pregnancy <20		0.84 (0.70-1.00)
Age at first pregnancy 20-24		0.80 (0.68-0.94)
Age at first pregnancy 25-29		0.95 (0.81-1.12)
Age at first pregnancy 30-34		1.02 (0.84-1.24)
Age at first pregnancy ≥35		1.38 (1.08-1.77)
Age at menopause		1.00 (1.00-1.003)
Family History		1.26 (1.14-1.40)
Postmenopausal status		1.04 (0.87-1.24)
Exercise		0.995 (0.99-1.00)
Alcohol		1.01 (1.00-1.01)
Age at menarche		0.99 (0.96-1.02)
Oophorectomy		0.89 (0.76-1.04)
Ethnic origin		0.94 (0.75-1.18)

Table S1b Type of HRT full adjusted model

HRT type		Age Adj.	Fully adj.
	**N%BC/N% no BC**	**HR (95% CI)**	**HR (95% CI)**
**Never**	998 (2.8)/ 34397 (97.2)	1.00	1.00
**Former oestrogen only**	191 (2.9)/ 6334 (97.1)	0.90 (0.76-1.05)	0.95 (0.79-1.14)
**Former combined**	316 (3.4)/ 9032 (96.6)	1.04 (0.91-1.19)	1.06 (0.93-1.22)
**Current oestrogen only**	63 (2.8)/ 2183 (97.2)	0.96 (0.74-1.98)	1.03 (0.79-1.34)
**Current combined**	95 (4.5)/ 1999 (95.5)	1.60 (1.30-1.98)	1.64 (1.32-2.03)

Age		1.02 (1.01-1.02)	1.02 (1.01-1.03)
BMI			1.23 (1.16-1.30)
Height			1.06 (1.03-1.10)
BMI20			0.77 (0.69-0.87)
HRTcurrent*BMI			0.83 (0.69-1.00)
HRTformer*BMI			0.91 (0.82-1.01)
BMI*BMI20			0.96 (0.90-1.02)
Age at first pregnancy <20			0.85 (0.70-1.02)
Age at first pregnancy 20-24			0.81 (0.69-0.95)
Age at first pregnancy 25-29			0.95 (0.81-1.12)
Age at first pregnancy 30-34			1.02 (0.84-1.24)
Age at first pregnancy ≥35			1.38 (1.08-1.76)
Age at menopause			1.00 (1.00-1.003)
Family History			1.27 (1.15-1.41)
Postmenopausal status			1.06 (0.89-1.26)
Exercise			0.995 (0.99-1.00)
Alcohol			1.008 (1.00-1.01)
Age at menarche			0.99 (0.96-1.02)
Oophorectomy			0.97 (0.81-1.15)
Ethnic origin			1.06 (0.85-1.33)

Table S1c HRT status and BC risk and ER+ve BC

		Age adj.	Fully adj.
HRT use status	**N% ER+ BC/ N% no BC**	**HR (95% CI)**	**HR (95% CI)**
**Never**	848 (2.4)/ 34942 (97.6)	1,00	1.00
**Former**	418 (2.7)/ 15212 (97.3)	0.99 (0.87-1.12)	1.05 (0.92-1.20)
**Current**	137 (3.1)/4251 (96.9)	1.33 (1.11-1.59)	1.45 (1.20-1.74)
Age		1.02 (1.01-1.03)	1.03 (1.02-1.04)
BMI			1.25 (1.17-1.33)
Height			1.07 (1.03-1.12)

BMI20	0.80 (0.71-0.91)
BMI*HRTcurrent	0.96 (0.92-1.00)
BMI*HRTformer	0.99 (0.97-1.01)
BMI20*BMI	0.92 (0.85-0.98)
Age at first pregnancy <20	0.86 (0.70-1.05)
Age at first pregnancy 20-24	0.81 (0.68-0.97)
Age at first pregnancy 25-29	0.97 (0.81-1.15)
Age at first pregnancy 30-34	1.07 (0.86-1.32)
Age at first pregnancy≥35	1.50 (1.15-1.96)
Age at menopause	1.00 (1.00-1.003)
Postmenopausal status	0.98 (0.81-1.18)
Family History	1.32 (1.18-1.47)
Exercise	0.99 (0.99-1.00)
Alcohol	1.01 (1.01-1.01)
Age at menarche	0.99 (0.96-1.03)
Oophorectomy	0.86 (0.72-1.02)
Ethnic origin	1.09 (0.85-1.38)

Units: age (1 year), BMI (5 BMI units), BMI at age 20 (5 BMI units), height (5 cm), age at menopause (1 year), exercise (1 hour per week), alcohol (1 unit per week), age at menarche (1 year)
