# Supplementary material for: Is Breast Cancer Risk Associated with Menopausal Hormone Therapy Modified by Current or Early Adulthood BMI or Age of First Pregnancy?
Source: Cancers (Basel). 2021 May 31;13(11):2710. doi: 10.3390/cancers13112710 (PMC8199436; doi:10.3390/cancers13112710)
Supplement: Supplementary file 1 [file cancers-13-02710-s001.zip › Table_S2.pdf]

Table S2a. HRT status and BC risk fully adjusted model including current BMI interaction term

HRT use status		Age adj.	Fully adj.
	**N%BC/N% no BC**	**HR (95% CI)**	**HR (95% CI)**
**Never**	998 (2.8)/ 34397 (97.2)	1.00	1.00
**Former**	507 (3.2)/ 15366 (96.8)	0.98 (0.88-1.10)	1.03 (0.91-1.17)
**Current**	158 (3.6)/ 4182 (96.4)	1.27 (1.07-1.50)	1.35 (1.13-1.60)
Age		1.02 (1.01-1.02)	1.02 (1.01-1.03)
BMI			1.23 (1.16-1.30)
Height			1.06 (1.02-1.10)
BMI20			0.77 (0.69-0.87)
HRTcurrent*BMI			0.81 (0.67-0.98)
HRTformer*BMI			0.91 (0.82-1.01)
BMI*BMI20			0.96 (0.90-1.02)
Age at first pregnancy <20			0.84 (0.70-1.01)
Age at first pregnancy 20-24			0.80 (0.69-0.94)
Age at first pregnancy 25-29			0.95 (0.81-1.12)
Age at first pregnancy 30-34			1.02 (0.84-1.24)
Age at first pregnancy ≥35			1.39 (1.08-1.77)
Age at menopause			1.00 (1.00-1.003)
Family History			1.27 (1.14-1.40)
Postmenopausal status			1.04 (0.87-1.24)
Exercise			0.995 (0.99-1.00)
Alcohol			1.008 (1.00-1.01)
Age at menarche			0.99 (0.96-1.02)
Oophorectomy			0.89 (0.76-1.05)
Ethnic origin			1.06 (0.85-1.33)

Table S2b. Fully adjusted model including BMI20 interaction term

HRT use status		
	**N%BC/N% no BC**	**HR (95% CI)**

**Never**	998 (2.8)/ 34397 (97.2)	1.00
**Former**	507 (3.2)/ 15366 (96.8)	1.03 (0.92-1.17)
**Current**	158 (3.6)/ 4182 (96.4)	1.35 (1.13-1.61)
Age		1.02 (1.01-1.03)
BMI		1.23 (1.16-1.31)
Height		1.06 (1.02-1.10)
BMI20		0.76 (0.66-0.87)
HRTcurrent*BMI		0.8 (0.65-0.99)
HRTformer*BMI		0.90 (0.80-1.00)
HRTcurrent*BMI20		1.05 (0.72-1.53)
HRTformer*BMI20		1.06 (0.85-1.33)
BMI*BMI20		0.96 (0.90-1.02)
Age at first pregnancy <20		0.84 (0.70-1.01)
Age at first pregnancy 20-24		0.80 (0.69-0.94)
Age at first pregnancy 25-29		0.95 (0.81-1.12)
Age at first pregnancy 30-34		1.02 (0.84-1.24)
Age at first pregnancy ≥35		1.39 (1.08-1.77)
Age at menopause		1.00 (1.00-1.003)
Family History		1.27 (1.14-1.40)
Postmenopausal status		1.04 (0.87-1.24)
Exercise		0.995 (0.99-1.00)
Alcohol		1.008 (1.00-1.01)
Age at menarche		0.99 (0.961.02)
Oophorectomy		0.89 (0.76-1.05)
Ethnic origin		1.06 (0.85-1.05)

Table S2c. HRT status and BC risk fully adjusted, including HRT use* current BMI * BMI20 interaction term

HRT use status

	N%BC/N% no BC	HR (95% CI)
**Never**	998 (2.8)/ 34397 (97.2)	1.00

**Former**	507 (3.2)/ 15366 (96.8)	0.99 (0.88-1.13)
**Current**	158 (3.6)/ 4182 (96.4)	1.34 (1.12-1.61)
Age		1.02 (1.01-1.03)
BMI		1.23 (1.16-1.31)
Height		1.06 (1.02-1.10)
BMI		1.24 (1.16-1.31)
BMI20		0.77 (0.66-0.89)
HRTcurrent*BMI		0.80 (0.65-0.98)
HRTformer*BMI		0.89 (0.79-1.00)
HRTcurrent*BMI20		1.04 (0.70-1.55)
HRTformer*BMI20		1.01 (0.79-1.29)
HRTcurrent*BMI*BMI20		0.99 (0.74-1.31)
HRTformer*BMI*BMI20		1.08 (0.94-1.23)
BMI*BMI20		0.94 (0.87-1.02)
Age at first pregnancy <20		0.84 (0.70-1.01)
Age at first pregnancy 20-24		0.80 (0.69-0.94)
Age at first pregnancy 25-29		0.95 (0.81-1.12)
Age at first pregnancy 30-34		1.02 (0.84-1.24)
Age at first pregnancy ≥35		1.38 (1.08-1.77)
Age at menopause		1.00 (1.00-1.003)
Family History		1.27 (1.14-1.40)
Postmenopausal status		1.04 (0.87-1.24)
Exercise		0.995 (0.99-1.00)
Alcohol		1.01 (1.00-1.01)
Age at menarche		1.00 (0.97-1.03)
Oophorectomy		0.89 (0.76-1.04)
Ethnic origin		0.94 (0.75-1.18)

3d Stratified analysis ER+

	BMI20 < 21.6kg/m2	BMI20 < 21.6kg/m2	BMI 20 ≥ 21.6kg/m2	BMI 20 ≥ 21.6kg/m2

	N% no BC/N% ER+BC	HR (95%CI)	% no BC/ N%ER+ BC	HR (95%CI)
**Never BMI < 26.4kg/m2**	9920 (97.9)/ 210 (2.1)	1,00	6276 (98.1)/119 (1.9)	0.89 (0.71-1.12)
**Never BMI ≥26.4kg/m2**	4610 (96.6)/163 (3.4)	1.68 (1.37-2.07)	4142 (97.5)/ 357 (2.5)	1.25 (1.05-1.49)
**Former BMI< 26.4kg/m2**	4329 (97.5)/ 111 (2.5)	1.10 (0.86-1.39)	2766 (97.4)/ 73 (2.6)	1.12 (0.84-1.47)
**Former BMI ≥ 26.4kg/m2**	2685 (97.0)/ 83 (3.0)	1.36 (1.05-1.78)	5874 (97.3)/165(2.7)	1.29 (1.04-1.61)
**Current BMI < 26.4kg/m2**	1409 (96.8)/47 (3.2)	1.66 (1.20-2.28)	822 (96.4)/ 31 (3.6)	1.87 (1.28-2.74)
**Current BMI ≥ 26.4kg/m2**	569 (96.3)/22 (3.7)	2.00 (1.29-3.12)	1453 (97.5)/38 (2.5)	1.34 (0.94-1.91)
