# Supplementary material for: Is Breast Cancer Risk Associated with Menopausal Hormone Therapy Modified by Current or Early Adulthood BMI or Age of First Pregnancy?
Source: Cancers (Basel). 2021 May 31;13(11):2710. doi: 10.3390/cancers13112710 (PMC8199436; doi:10.3390/cancers13112710)
Supplement: Supplementary file 1 [file cancers-13-02710-s001.zip › Table_S3.pdf]

Table S3 characteristics of women with known and unknown HRT status

	HRT Status	
	Total	Unknown
**Number of women**	56489	514
Ethnicity		
White	53571 (94.8)	460 (89.5)
Other	2918 (5.2)	54 (10.5)
Age at study entry (years)		
Median (IQR)	56.9 (51.6-63.6)	54.7 (51.0-63.2)
Mean (SD)	57.8 (7.0)	57.1 (7.4)
Menopausal status		
premenopausal and perimenopausal	18764 (33.2)	217 (42.3)
postmenopausal	37725 (66.8)	297 (57.8)
Age at menopause		
Median (IQR)	50.0 (46.0-53.0)	50.0 (48.0-55.0)
Mean (SD)	49.0 (5.6)	49.7 (5.3)
Age at menarche		
Median (IQR)	13.0 (12.0-14.0)	13.0 (12.0-14.0)
Mean (SD)	12.9 (1.6)	13.0 (1.6)
BMI		
Underweight or normal weight	20216 (35.9)	136 (26.6)
Overweight	22249 (39.5)	234 (45.7)
Obese	13808 (24.6)	142 (27.7)
Median (IQR)	26.4 (23.8-29.9)	26.4 (24.6-30.8)
Mean (SD)	27.4 (5.4)	28.2 (6.0)
BMI at age 20		
Underweight or normal weight	50119 (88.8)	451 (87.7)
Overweight	5105 (9.0)	45 (8.8)
Obese	1243 (2.2)	18 (3.5)
Median (IQR)	21.6 (20.3-23.0)	21.6 (20.3-23.0)
Mean (SD)	22.0 (3.1)	22.0 (3.1)
Median Height (m) (IQR)	1.62 (0.07)	1.63 (1.57-1.65)
Mean Height (m) (SD)	1.63 (1.57-1.65)	1.62 (0.07)
Oophorectomy		
Yes	6696 (12.0)	45 (8.8)

No	49693 (88.0)	469 (91.2)
VAS Density		
Median (IQR)	24.8 (14.9-35.8)	23.3 (15.1-34.0)
Mean (SD)	27.1 (16.2)	26.3 (15.7)
Family History of Breast or Ovarian Cancer		
Yes	15738 (27.9)	145 (28.2)
No	40748 (72.1)	369 (71.8)
Median exercise hours per week (IQR)	3.5 (1.5-7.0)	3.5 (2.0-5.0)
Median alcohol units per week (IQR)	4.0 (5.0-10.0)	4.0 (0.0-8.0)
Parity and age at first pregnancy		
Nulliparous	7330 (13.0)	71 (13.8)
<30	41408 (73.3)	382 (74.3)
≥30	7751 (13.7)	61 (11.9)
Median age at first pregnancy (IQR)	24.0 (21.0-28.0)	23.0 (20.0-27.0)
Mean age at first pregnancy (SD)	24.4 (5.3)	23.8 (5.0)
Median EIMD score 2010 (IQR)	18.9 (10.4-35.1)	25.5 (12.3-43.6)
