# Supplementary material for: Is Breast Cancer Risk Associated with Menopausal Hormone Therapy Modified by Current or Early Adulthood BMI or Age of First Pregnancy?
Source: Cancers (Basel). 2021 May 31;13(11):2710. doi: 10.3390/cancers13112710 (PMC8199436; doi:10.3390/cancers13112710)
Supplement: Supplementary file 1 [file cancers-13-02710-s001.zip › Table_S4.pdf]

Table S4 HRT status and BC risk fully adjusted with interaction term for age of first pregnancy

	N% no BC/N % no BC	HR	95%CI
**Never**	998 (2.8)/ 34397 (97.2)	1	
**Current**	158 (3.6)/ 4182 (96.4)	1,34	1.06-1.70
**Former**	507 (3.2)/ 15366 (96.8)	0,93	0.79-1.09
Age		1,02	1.01-1.03
HRTcurrent*Age at First Pregnancy		1,00	0.98-1.02
HRTformer*Age at First Pregnancy		0,99	0.98-1.00
Age at first pregnancy		1,00	1.00-1.01
Oophorectomy		0,88	0.75-1.03
Family History		1,26	1.14-1.40
BMI		1,16	1.11-1.23
BMI20		0,77	0.69-0.86
BMI*BMI20		0,97	0.91-1.03
Height		1,07	1.03-1.11
Age at Menopause		1,00	1.00-1.003
Age at Menarche		0,99	0.96-1.02
Exercise		0,99	0.99-1.00
Alcohol		1,01	1.00-1.01
Postmenopausal Status		1,04	0.87-1.23
Ethnic origin		1,07	0.86-1.33
